# Factors related to the improvement in quality of life for depressed inpatients treated with fluoxetine

**DOI:** 10.1186/s12888-017-1471-3

**Published:** 2017-08-25

**Authors:** Wei-Cheng Yang, Ching-Hua Lin, Fu-Chiang Wang, Mei-Jou Lu

**Affiliations:** 1 Kaohsiung Municipal Kai-Syuan Psychiatric Hospital, No.130, Kaisyuan 2nd Road, Lingya District, Kaohsiung City, 80276 Taiwan; 2 Department of Psychiatry, Faculty of Medicine, College of Medicine, Kaohsiung Medical University, No.100, Shihcyuan 1st Road, Sanmin District, Kaohsiung City, 80708 Taiwan

## Abstract

**Background:**

The aim of this study was to explore the relationships between depressive symptoms and health-related quality of life (HRQOL) measurements for inpatients with major depressive disorder (MDD) before and after 6-week fluoxetine treatment, and to elucidate the factors related to the HRQOL changes.

**Methods:**

A total of 131 inpatients with MDD were enrolled to receive 20 mg of fluoxetine for 6 weeks. Symptom severity and adverse events were assessed at weeks 0, 1, 2, 3, 4, and 6 using the 17-item Hamilton Depression Rating Scale (HAMD-17) and UKU Side Effect Rating Scale, respectively. HRQOL was measured using the Short Form 36 (SF-36), including 8 subscales, physical component summary (PCS) and mental component summary (MCS), at baseline and week 6. Spearman’s coefficient, Cohen’s d, and multiple linear regression model were used for statistical analysis.

**Results:**

One hundred and six patients completing all measures at weeks 0 and 6 entered the analysis. HAMD-17 negatively correlated with SF-36 at baseline and week 6. The HAMD-17 had a larger effect size than SF-36. MCS, rather than PCS, showed statistically significant improvement. After using multiple linear regression analysis, age at onset, HAMD-17 score change, and number of adverse events reported during the trial period were related to MCS change after adjusting for confounding variables.

**Conclusions:**

Fluoxetine treatment was associated with an improvement in depressive symptomology and HRQOL. Depressive symptoms had a greater extent of change than HRQOL. Clinicians must consider the negative effects of adverse events caused by antidepressants on the improvement of HRQOL.

**Trial registration:**

http://clinicaltrials.gov, NCT01075529, retrospectively registered 24/2/2010.

## Background

Health-related quality of life (HRQOL) has been defined as “those aspects of self-perceived well-being that are related to or affected by the presence of disease or treatment [1].” The assessment of HRQOL should consider patients’ subjective views of their life circumstances [2], including perceptions of social relationships, physical health, mental heath, functioning in daily activities and work, and an overall sense of well-being [3]. Compared to the general population and patients with other chronic diseases, such as diabetes, arthritis or cardiovascular disease, HRQOL is substantially reduced in patients suffering from major depressive disorder (MDD) [4, 5]. Depressed patients typically regard their well-being, social functioning and living conditions as being worse than they appear to independent observers [6].

There is an increasing demand to adopt HRQOL as an outcome measure for clinical trials and health care policy [7]. HRQOL measurement should consider patients’ subjective views of their life circumstances [8]. This may provide a more extensive assessment of treatment outcomes than those based solely on improvement in symptoms of depression. For example, while antidepressant medication may decrease depressive symptoms, it may also induce unpleasant adverse events that impair the patient’s HRQOL [9]. Souetre et al. [10] conducted a trial to compare the effect of fluoxetine vs. tricyclic antidepressants (TCAs) on HRQOL of patients with MDD. Although depressive symptoms were comparable for the 2 groups, the fluoxetine group had HRQOL superior to that of the TCAs group after adjusting for confounding variables. The authors concluded that one of the reasons for a superior level of HRQOL may have been due to differences in drug-related adverse events. Therefore, a patient’s perception of health and well-being is not associated with symptom reduction alone. HRQOL could be regarded as a kind of risk-benefit analysis. The American Psychiatric Association guideline for the treatment of patients with MDD [11] suggests that decreases in depressive symptoms and improvement in HRQOL are recognized as the goals in treating depressed patients. In essence, effective treatment should lead to symptom reduction and improvement in HRQOL.

To date, there is still no study to investigate the level of HRQOL change after treatment for depressed patients negatively impacted by adverse events occurred during the trial period. The aim of this study was to explore the relationships between depressive symptoms and HRQOL measurements for MDD inpatients before and after a 6-week fluoxetine treatment, and to elucidate the factors related to the HRQOL changes.

## Methods

### Subjects

The current study was part of a clinical trial, documented elsewhere [12]. The trial was approved by Kai-Syuan Psychiatric Hospital’s institutional review board and conducted in accordance with both Good Clinical Practice procedures and the most recent revision of the Declaration of Helsinki. Written, informed consent was obtained from all participants after a full explanation of study aims and procedures. This study was registered on http://clinicaltrials.gov (Identifier number: NCT01075529).

As previously described in detail [12], all MDD patients newly hospitalized for acute treatment were screened and evaluated by three board-certified psychiatrists using the Structured Clinical Interview for DSM-IV [13] to ensure diagnostic accuracy. Han Chinese patients in Taiwan were enrolled in this study if they: 1) were physically healthy with normal laboratory tests (including electrocardiography and chest X-ray), 2) were aged 18–70 years, and 3) satisfied DSM-IV criteria for MDD. The exclusion criteria were: 1) a baseline score of 17-item Hamilton Depression Rating Scale (HAMD-17) [14] < 18, 2) a Clinical Global Impression of Severity (CGI-S) [15] < 4, 3) psychotic depression, bipolar I or II disorder, schizophrenia, or any other psychotic disorder, 4) a DSM-IV diagnosis of substance abuse or dependence (including alcohol) within the past 6 months, 5) mental disorders due to organic factors, 6) severe cognitive impairment, 7) initiating or ending formal psychotherapy within six weeks prior to enrollment, 8) receiving formal psychotherapy during the trial period, 9) Treatment-resistant depression was defined as a lack of response to 2 or more adequate trials of different classes of antidepressants [16]. An adequate trial is defined as 4–6 weeks of treatment with an antidepressant at a dosage considered therapeutic [17], 10) a history of poor response to fluoxetine (defined as no obvious clinical improvement after receiving at least 20 mg/day of fluoxetine for 4 weeks as reported by medical record, the patients, or their family) or intolerance to fluoxetine, 11) a history of electroconvulsive therapy, and 11) pregnancy or lactation.

### Procedures and assessments

After a washout period of at least 72 h, patients received open-label fluoxetine treatment at a fixed dose of 20 mg daily for 6 weeks. During the course of treatment, psychiatrists had the option of adding certain anxiolytic and/or sedative-hypnotic medications for brief periods, based on clinical necessity. No other psychotropic agents were used at bedtime to treat insomnia. Drug adherence was monitored and ensured by psychiatric nurses. Demographic and clinical characteristics of the participants were gathered at baseline. Age at onset was regarded as the age at which the first major depressive episode occurred.

Depression severity was assessed at baseline, and again at weeks 1, 2, 3, 4, and 6 by three board-certified psychiatrists using the HAMD-17. Inter-rater reliability of HAMD-17 was analyzed with the ANOVA test. The intraclass correlation coefficient of reliability during pre-study training was 0.95 among the raters for assessing 10 patients. To maintain high interrater reliability and prevent rater drift, raters met at least once a month for training and reliability retesting.

Adverse-event burden was assessed by the Utvalg for Kliniske Undersogelser Side Effect Rating Scale (UKU) [18] and by the registration of adverse events at baseline and at weeks 1, 2, 3, 4, and 6. UKU is a clinician-rated scale with 48 items which include psychic subscale, neurological subscale, autonomic subscale, and other subscale. UKU contains a Likert scale of 0–3 for degree of severity, and is commonly used to assess the tolerability of antidepressants in clinical trials [19, 20]. A score of 1, 2 or 3 on any UKU item that first occurred or worsened during treatment indicates an adverse event. Therefore, adverse events included those absent at baseline and the worsening of an adverse event, already present at baseline, during the trial [21]. The number of adverse events reported during the trial period is used to determine the adverse-event burden.

HRQOL measurement included Medical Outcomes Study Short-Form-36 (SF-36) [22] at baseline and week 6. The SF-36 is comprised of the physical component summary (PCS) which measures physical health, and the mental component summary (MCS) which measures mental health. PCS includes 4 subscales: 1) physical functioning, 2) role physical limitations, 3) body pain, 4) general health; MCS includes 4 subscales: 1) vitality, 2) social functioning, 3) role emotional limitations, and 4) mental health. SF-36 raw scores were processed according to the user manual to obtain standardized scores for all 8 SF-36 subscales, PCS and MCS [23]. Scores for the 8 SF-36 subscales range from 0 to 100, with a higher score representing better HRQOL. The PCS and MCS were standardized according to general population means and variances to produce scores with a common mean of 50 and standard deviation of 10 (T-scores). Thus, any score < 50 represents a reduction from “normal” health. At the moment no Taiwanese population data on the SF-36 exists, therefore the population mean and standard deviation are from the 1998 general U.S. population [24]. The Taiwanese version of the SF-36 shows good validity and reliability [25] and in the current sample the reliabilities were estimated at 0.78 (week 0) and 0.90 (week 6). Since the SF-36 is a self-reported questionnaire. For illiterate patients, SF-36 was completed during face-to-face interviews.

### Statistical analysis

The data were analyzed using IBM SPSS Statistics for Windows, version 20. Statistical significance was set at *p* < 0.05. Descriptive statistics were listed for the demographic and clinical variables of subjects.

Spearman’s coefficient was used to examine the correlations between HAMD-17 and SF-36 (i.e., scores of the 8 subscales, PCS, and MCS) at week 0 and again at week 6. Pre-test and post-test HAMD-17 and SF-36 scores were compared using paired t-test. Cohen’s d [26] was computed as a measure of the pre-post effect sizes for HAMD-17 and SF-36. Effect size was defined as the mean of difference between baseline and post-treatment scores for each measure divided by the standard deviation of difference [27]. A d-value of 0.20 indicates a small effect size, 0.50 medium effect size, and 0.80 large effect size [26]. Effect size provides an estimate of the magnitude of between-group differences on a standard scale. Large effect sizes demonstrate clinically relevant improvement at the end point. Effect size is a unitless measure, thus appropriate for comparisons involving scales with different metrics [28]. Therefore, the degrees of improvement could be compared among the different subscales.

A multiple linear regression model was used to explore the variables associated with HRQOL changes as measured by PCS or MCS. The multiple linear regression model was bootstrapped (bootstrap samples = 1000) to obtain statistically more robust results for the relatively small sample size. These variables included sex, age, age at onset, number of previous depressive episodes, baseline PCS or MCS, HAMD-17 score changes, and number of adverse events reported during the trial period. Variance Inflation Factor (VIF) values larger than 10 were regarded as having presence of multicollinearity [29].

## Results

**Fig. 1:**
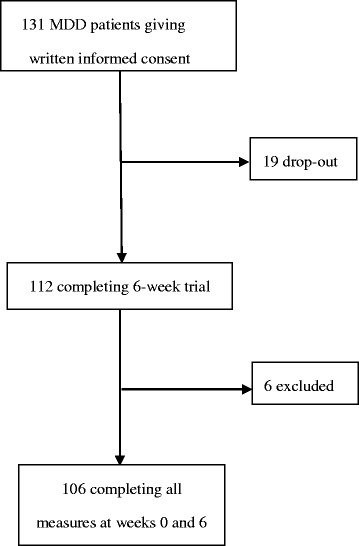
Selection of participants for the analysis

A total of 131 acutely ill MDD inpatients was enrolled. One hundred and six (80.9%) of 131 patients who completed all measures at baseline and week 6 were included in the analysis. The participant selection process is shown in Fig. 1. There were no statistical significances between the patients included (*n* = 106) and those without (*n* = 25) with respect to sex (*p* = 0.276), age (*p* = 0.219), age at onset (*p* = 0.110), number of previous episodes (*p* = 0.491), and baseline HAMD-17 (*p* = 0.733) (data not shown in the Table). Table 1 lists the demographic and clinical characteristics of the subjects included. The mean HAMD-17 ± SD of score of 31.4 ± 6.4 at baseline reflected fairly severely depressed subjects. The mean of number of adverse events reported during the trial period was 4.6 ± 3.6. The top three categorical adverse events were dizziness (*n* = 42, 39.6%), reduced salivation (*n* = 40, 37.7%), and polyuria/polydipsia (*n* = 33, 31.1%).

**Table 1:** Demographic and clinical variables of subjects (*n* = 106) at baseline

Variables	Distribution
Sex-male, %	23 (21.7)
Age, mean (SD), y	45.8 (11.2)
Age at onset, mean (SD), y	39.6 (12.0)
Number of previous episodes, mean (SD)	2.5 (1.9)
Number of adverse events, mean (SD)	4.6 (3.6)
HAMD-17 ^a^ score, mean (SD)	31.4 (6.4)

^a^HAMD-17 = 17-item Hamilton Depression Rating Scale

HAMD-17 scores negatively correlated with 8 subscales, PCS, and MCS of SF-36, regardless of whether at baseline or at week 6 (Table 2). Additionally, these correlations improved markedly at week 6.

**Table 2:** Correlations between HAMD-17 and SF-36 for both instruments measured at week 0 and measured at week 6 (*n* = 106)

	HAMD-17 at week 0		HAMD-17 at week 6
SF-36 at week 0		SF-36 at week 6	
Physical functioning	*r* = −0.313^**^	Physical functioning	*r* = −0.582^**^
Role physical limitations	*r* = −0.251^**^	Role physical limitations	*r* = −0.433^**^
Bodily pain	*r* = −0.208^*^	Bodily pain	*r* = −0.484^**^
General health	*r* = −0.390^**^	General health	*r* = −0.713^**^
Vitality	*r* = −0.413^**^	Vitality	*r* = −0.699^**^
Social functioning	*r* = −0.211^*^	Social functioning	*r* = −0.609^**^
Role emotional limitations	*r* = −0.198^*^	Role emotional limitations	*r* = −0.493^**^
Mental health	*r* = −0.377^**^	Mental health	*r* = −0.662^**^
Physical component summary	*r* = −0.311^**^	Physical component summary	*r* = −0.569^**^
Mental component summary	*r* = −0.289^**^	Mental component summary	*r* = −0.657^**^

^a^HAMD-17 = 17-item Hamilton Depression Rating Scale

^b^SF-36 = Medical Outcomes Study Short-Form-36

*p < 0.05, **p < 0.01

HAMD-17 and SF-36 at baseline and week 6 are listed in Table 3. HAMD-17 decreased significantly from 31.4 ± 6.4 to 13.7 ± 8.3. Compared to Taiwanese norms [25], baseline measures of HRQOL were significantly impaired. Among 8 SF-36 subscales, improvements ranged from an increase of 2.4 ± 23.7 points in the physical functioning subscale, and an increase of 17.6 ± 40.0 points in the role emotional limitations subscale. However, 8 SF-36 subscales after the 6-week treatment were still lower than those of Taiwanese norms. All SF-36 subscales, but not physical functioning, improved significantly after treatment. The greatest improvements were found in the subscales measuring mental health (i.e., mental health, role emotional limitations, and vitality) as opposed to physical health (i.e., physical functioning, role physical limitations, and body pain). The observed improvement of PCS was only minimal, but this improvement was not statistically significant. The MCS showed statistically significant improvement after the 6-week treatment (Table 3). The pre-post effect sizes for PCS and MCS were 0.142 and 0.548, respectively, indicating that fluoxetine treatment was more strongly associated with improvements in mental health than physical health.

**Table 3:** Pre-post comparisons of HAMD-17^a^ and SF-36^b^ for patients completing the 6-week treatment (*n* = 106)

Measure	Taiwanese norms (Mean ± SD)	Week 0 (Mean ± SD)	Week 6 (Mean ± SD)	Difference (Mean ± SD)	p	Effect size
HAMD-17		31.4 ± 6.4	13.7 ± 8.3	−17.7 ± 8.5	<0.001*	−2.093
SF-36						
Physical functioning	92.24 ± 16.16	61.5 ± 25.7	63.9 ± 25.4	2.4 ± 23.7	0.299	0.101
Role physical limitations	83.65 ± 33.27	15.1 ± 30.8	28. 5 ± 41.9	13.4 ± 45.1	0.003*	0.298
Bodily pain	84.84 ± 19.42	45.3 ± 26.1	50.9 ± 29.2	5.6 ± 24.1	0.018*	0.233
General Health	69.29 ± 21.27	28.0 ± 22.5	35.9 ± 26.4	7.9 ± 22.8	<0.001*	0.348
Vitality	68.27 ± 18.66	24.6 ± 19.6	33.7 ± 23.2	9.2 ± 21.9	<0.001*	0.417
Social functioning	86.81 ± 17.05	33.8 ± 22.9	43.0 ± 26.2	9.2 ± 27.2	<0.001*	0.338
Role emotional limitations	79.40 ± 36.07	5.7 ± 17.5	23.3 ± 39.1	17.6 ± 40.0	<0.001*	0.441
Mental health	73.01 ± 16.55	26.7 ± 19.3	37.6 ± 22.2	10.9 ± 23.9	<0.001*	0.457
Physical component summary		39.3 ± 10.0	40.6 ± 10.6	1.3 ± 9.3	0.148	0.142
Mental component summary		23.3 ± 8.62	29.7 ± 11.3	6.4 ± 11.6	<0.001*	0.548

^a^HAMD-17 = 17-item Hamilton Depression Rating Scale

^b^SF-36 = Medical Outcomes Study Short-Form-36

*Statistically significant

Consequently, a multiple linear regression model using the bootstrap method was applied to analyze only the predictors related to MCS change during treatment. Age at onset of major depressive episode, HAMD-17 score change, and the number of adverse events were significant predictors associated with MCS change after adjusting for sex, age, number of previous episodes, and baseline MCS. All VIFs were less than 10, and indicated no presence of multicollinearity in the model (Table 4). The non-standardized regression weights (B) in Table 4 require interpretation. For example, after adjusting for other predictors, patients increased likelihood of later age at onset of illness of having an increased MCS score (per year increased in age at onset they gained 0.283 points in MCS).

**Table 4:** Multiple linear regression model with the changes of mental component summary (MCS) as a dependent variable (*n* = 106)

Dependent variable	Independent variable	B^a^	SE^b^	p	Adjusted R^2^	VIF^c^
MCS change	Sex	0.681	2.195	0.756	0.434	1.097
	Age	−0.019	0.105	0.850		2.565
	Age at onset	0.283	0.107	0.006*		2.949
	Number of previous episodes	0.151	0.549	0.783		1.424
	HAMD-17 score change	−0.443	0.118	0.001*		1.160
	Number of adverse events	−0.615	0.238	0.008*		1.197
	Baseline MCS	−0.570	0.115	0.001*		1.032

^a^B = unstandardized regression coefficient

^b^SE = bootstrap standard error

^c^VIF = Variance Inflation Factor

*Statistically significant

## Discussion

The first main finding of the current study was that symptomatic changes only accounted for a limited amount of variance in HRQOL changes. The second was that being younger at onset and adverse events during treatment hindered improvement in HRQOL. Our results replicated previous findings, that treating MDD inpatients with antidepressants is efficacious in relieving depression symptoms and improving HRQOL [30, 31].

Similar to results seen in other studies [5, 9, 32], there was an inversely significant correlation between depression severity and HRQOL measurements at baseline and again at week 6 (Table 2). Table 2 also shows that the correlations between baseline HAMD-17 and SF-36 (i.e., 8 subscales, PCS and MC) increased after 6 weeks of treatment. One possibility is that since patients responded differently to treatment, the variation in scores got larger during treatment, and therefore, even if the population covariances between measures did not change at all, the correlations consequently may increase. Another is that severity of illness also affected the level of correlation. Patients with severe illness may have difficulty completing the self-reported questionnaire (e.g., SF-36) on their own, while their self-perception and cognitive distortion may not reflect reality for rating scales [33–35]. Therefore, depressed patients with severe symptomatology (e.g., at baseline) may underestimate their HRQOL [34]. Clinicians tend to recognize severe illness based on non-verbal evidence [36]. As patients’ conditions improve after treatment, their verbal reports become more important for less severe illness, because they are more capable of clearly identifying their problems [35].

Improvements varied in magnitude measured by effect sizes across SF-36 subscales, with the greatest improvements observed in the subscales that focus more on mental health as opposed to physical health (Table 3). Table 3 also shows that physical functioning score was rather high, but the role physical limitations score, body pain score, and general health score were low. There are several explanations. First, all participants were physically healthy with normal laboratory tests. A growing body of evidence suggests that pain and depression may operate within similar areas of the brain that regulate both mood and the affective components of pain [37]. Depression and pain may interact bidirectionally [38–40], therefore it is reasonable that physical functioning was rather high in comparison to the other scores, and body pain was negatively affected by depression severity. Second, a moderate correlation existed between role physical limitations and role emotional limitations before (*r* = 0.458, ***p*** **< 0.001**) and after trial (*r* = 0.676, ***p*** **< 0.001**). Some severely depressed patients may have troubles realizing whether their reduction of working/activities duration came from physical difficulties (i.e., items for role physical limitations) or from emotional difficulties (i.e., items for role emotional limitations). Third, using the items of SF-36 (i.e., 11a. 11b, 11c, and 11d) to measure general heath only focuses on patients’ health conditions rather than highlighting mental health. The mean (SD) scores of item 17 of HAMD-17 (i.e., insight) before and after the trial were 0.5 (0.6) and 0.2 (0.4), respectively (data not shown in the Table). This suggests that most of the patients acknowledged being ill and unhealthy, thereby leading the low score in general health. However, another Taiwanese study (*n* = 95) [41] used the 8 subscales of SF-36 to measure HRQOL for MDD outpatients receiving 75 mg daily of venlafaxine before and after 1-month treatment. Their results were comparable to our findings, that the score of physical functioning (72.5 ± 20.9 to 82.7 ± 17.8) was rather high, but the scores for the role physical limitations (24.7 ± 34.1 to 55.3 ± 39.3), body pain (47.6 ± 19.2 to 62.2 ± 21.6), and general health (31.3 ± 17.4 to 47.4 ± 20.7) were low. Compared to a study [42] conducted in the U.S. for depressed patients treated with 20 mg daily of fluoxetine for 3 months, the mean score of physical functioning (73.2 to 75.8) was highest, but the mean score of vitality was very low (28.8 to 50.5). These results are similar to ours. However, the mean scores of role physical limitations (63.5 to 73.6) and general health score (57.7 to 63.3) were not too low, which indicates that cultural and ethnic factors should be explored in further studies. Symptom improvements rated by HAMD-17 had larger effect sizes (absolute value = 2.093) than SF-36 (Table 3). This means that depressive symptoms had a greater extent of change than HRQOL after the 6-week treatment [7]. This finding was also in accordance with other studies [43–45]. Furthermore, this finding was highly in line with theoretical models of response in mental health, especially the “phase model” proposed by Howard et al. [46], which posits that first a general hope factor causes a response similar to a placebo effect, then specific symptoms change and anything connected to the interpersonal and real life setting of the patients responds quite a bit later. Health-related quality of life can be seen as one indicator of this last area/ phase of treatment response. However, SF-36 in the subjects after treatment was still lower than that of Taiwanese norms (Table 3). McCall et al. [47] concluded that maximal improvement in HRQOL may lag weeks or months behind maximum symptom improvement. Therefore, HRQOL enhancement therapies, e.g., combinations of pharmacotherapy with psychotherapy [48], were still necessary after the trial.

SF-36 summary scores showed that MCS rather than PCS improved significantly after treatment. Our results were also consistent with previous antidepressant studies showing that PCS scores do not, or only marginally, change with time for depressed patients treated with antidepressant agents [45, 49, 50]. As MDD is a mental health problem, it is rational to assume that the mental health subscales improve more (i.e., greater effect sizes) than do physical health subscales after treatment. Physical functioning is highly correlated with PCS, and mental health correlates highly with MCS. MCS is designed to provide a valid summary of information contained in the 8 SF-36 subscales for both mental health status and changes over time. MCS has been established as a sensitive outcome measure in studies of clinical depression [51].

In the multiple linear regression model, greater MCS improvement after the 6-week fluoxetine use could be predicted for subjects with older age onset of MDD (Table 4). This result is consistent with the study by Moses et al. [52]. Early-onset MDD has been reported to represent developmental pathways characterized by a distinct set of sociodemographic and clinical features [53, 54], and is associated with a higher level of comorbidity and impairment, as well as significant genetic loading [55]. The greater improvement of subjects with older age onset of MDD may be due to their having developed better psychosocial contexts for quick improvement in HRQOL [52].

Previous studies [56, 57] have concluded that improvement in HRQOL correlates highly with improvement in depressive symptoms. As expected, the current study shows that the amelioration of depressive symptoms measured by HAMD-17 was still accompanied by improvements in MCS after adjusting for other variables (Table 4).

Adverse events due to antidepressants are widely believed to have a negative impact on HRQOL [49, 58]. Our results reveal that the number of adverse events reported during the trial period was negatively related to patients’ MCS improvement. This finding was comparable to that of other studies, in which antidepressants’ adverse events are associated with decreased HRQOL [59, 60]. Adverse events subsequent to fluoxetine use have been reported to be relatively mild and usually begin early in the course of therapy [61]. The SF-36 is designed to assess the HRQOL over the previous 4 weeks [22], so it is reasonable that SF-36 measurements cover the early course of fluoxetine treatment. In the present study, the number of adverse events is only negatively correlated with changes of mental health subscales (*r* = −0.196, ***p*** **= 0.044** for role emotional limitations to *r* = −0.327, ***p*** **= 0.001** for mental health) rather than physical health subscales (data not shown in the Table). Adverse events are supposed to generate psychological distress rather than physical injury to patients, and therefore influence mental health much more than physical health. Actually, no severe adverse events were reported during the trial period. If we excluded the number of adverse events from the multiple linear regression model, the adjusted R^2^ changed from 0.434 (Table 4) to 0.406 (data not shown in the Table). However, adverse events could be decreased with appropriate intervention [62]. In short, the number of adverse events could be considered a modifiable risk factor. Although the influence of adverse events on HRQOL is relatively mild; it should not be neglected. Fluoxetine might be a two-edged sword in terms of HRQOL improvement for patients with MDD. Adverse events may compromise patients’ HRQOL improvement, which may further compromise treatment adherence. Psychiatrists must help patients identify and manage these adverse events.

In the present study, multiple linear regression model (Table 4) explained approximately 43.4% of the variance (adjusted R^2^ = 43.4%) of MCS improvement, suggesting that improvement in depressive symptoms may not accurately represent improvement in HRQOL. The mechanism affecting improvement in HRQOL for patients with MDD is complex and multifactorial. Besides age at onset, symptom improvement, and number of adverse events, other potential variables related to HRQOL improvement require further exploration in future studies.

There were some limitations to the method of interpreting the results. First, use of a single site with a relatively small sample size limited the generalizability of our results. Second, assessments were performed during the acute stage, and the stability of these findings was not evaluated through time. The study period of 6 weeks was relatively short when considering the total duration of depression. However, it was fairly long, and sufficient for inpatient trials to detect initial antidepressant responses. Third, there are other HRQOL scales available for subjects, and different scales may yield different results. Fourth, this was an open-label design without a control group. Because of this, it was difficult to establish the degree to which clinical improvements were due to fluoxetine treatment, placebo effect, other psychiatric interventions, or the natural course of MDD. For example, hospitalization itself can be a significant non-pharmacological therapeutic factor affecting clinical improvement in patients with MDD. Therefore, this study did not establish a causal link between fluoxetine and any changes observed, since no control group was presented. Although, without a placebo-controlled group, the impact of placebo effects on the current study could not be estimated, it is unlikely the clinical response was solely attributable to placebo effects for the following reasons: first, the response (i.e., a reduction of 50% or more of the HAMD-17) rate (= 56.9%) was too high to be accounted for by the typical placebo effects, i.e., around 30%, as estimated from past clinical placebo-controlled antidepressant trials [63]; second, it has been demonstrated that patients with more severe depression are less susceptible to placebo effect [64]. However, a meta-analysis study [65] demonstrates that drug–placebo differences in antidepressant efficacy increase as a function of baseline severity, but are relatively small even for severely depressed patients. Finally, our sample was comprised of the most severely affected MDD patients who required hospitalization. Also, inpatients’ clinical situation is not very representative of outpatients.

## Conclusions

Our study showed that for hospitalized patients with MDD, fluoxetine improved not only depressive symptoms but also most aspects of HRQOL. Within the 6-week trial period, depressive symptoms showed a greater degree of change than HRQOL measurement. Age at onset of major depressive episode, HAMD-17 score change, and the number of adverse events experienced during the trial period were associated with MCS improvements after adjusting for sex, age, number of previous episodes, and baseline MCS. The regression model accounted for approximately 43.3% of the variance in MCS improvement. Further study is required for determining the long-term improvement in HRQOL, and the efficacy of other treatment modalities in improving HRQOL.

## Abbreviations

CGI-S: Clinical Global Impression of Severity
HAMD-17: 17-item Hamilton Depression Rating Scale
HRQOL: Health-related quality of life
MCS: Mental component summary
MDD: Major depressive disorder
PCS: Physical component summary
SF-36: Medical outcomes study short-form-36
TCAs: Tricyclic antidepressants
UKU: Utvalg for Kliniske Undersogelser
